# Strengthening of Porcine Plasma Protein Superabsorbent Materials through a Solubilization-Freeze-Drying Process

**DOI:** 10.3390/polym13050772

**Published:** 2021-03-03

**Authors:** Estefanía Álvarez-Castillo, Carlos Bengoechea, Antonio Guerrero

**Affiliations:** Escuela Politécnica Superior, Chemical Engineering Department, University of Seville, Calle Virgen de África, 7, 41011 Sevilla, Spain; cbengoechea@us.es (C.B.); aguerrero@us.es (A.G.)

**Keywords:** plasma protein, superabsorbent, protein-based material, freeze-drying, injection molding

## Abstract

The replacement of common acrylic derivatives by biodegradable materials in the formulation of superabsorbent materials would lessen the associated environmental impact. Moreover, the use of by-products or biowastes from the food industry that are usually discarded would promote a desired circular economy. The present study deals with the development of superabsorbent materials based on a by-product from the meat industry, namely plasma protein, focusing on the effects of a freeze-drying stage before blending with glycerol and eventual injection molding. More specifically, this freeze-drying stage is carried out either directly on the protein flour or after its solubilization in deionized water (10% *w*/*w*). Superabsorbent materials obtained after this solubilization-freeze-drying process display higher Young’s modulus and tensile strength values, without affecting their water uptake capacity. As greater water uptake is commonly related to poorer mechanical properties, the proposed solubilization-freeze-drying process is a useful strategy for producing strengthened hydrophilic materials.

## 1. Introduction

Superabsorbent materials are capable of absorbing and retaining water in quantities higher than ten times their own dry weight [1,2]. Traditionally, superabsorbent materials are based on acrylic derivatives [3,4], which are expensive, toxic, and highly pollutant due to their fairly low biodegradability. These materials are extensively used in the personal care industry, so their use time is relatively short as they are easily disposable, which contributes to the environmental issues caused by poorly biodegradable synthetic plastics. In contrast, some studies have pointed out the feasibility of obtaining superabsorbent materials from natural sources such as porcine plasma protein [5,6,7], soy protein [8,9,10,11], or gluten [12].

The meat industry produces a huge amount of blood, which is rich in proteins [13] and should not be directly disposed of in landfills or effluents due to its high organic charge, which can produce high pollution levels due to the high biochemical oxygen demand (BOD) [14,15,16]. Therefore, extensive use and revalorization of this by-product would be of great interest to increase the competitiveness and business growth of the meat industry and to promote the integration of circular economy principles. Plasma is the blood fraction that remains after the separation of the red cells and platelets [17] through centrifugation, which can be dried to obtain a porcine plasma protein (PPP) powder. This by-product is already used in the food industry either as an emulsifier, as a water-holding agent [18] in frankfurters [19,20] and sausages [13], or as an alternative to certain other protein ingredients, such as egg [21,22]. Furthermore, the excellent film-forming potential displayed by porcine plasma protein has proven to be useful in the development of films for food packaging, replacing synthetic plastics [23,24,25]. As mentioned earlier, some studies have also pointed out the potential of PPP for superabsorbent applications, showing water uptake capacity values as high as 3600% [6], which can be attributed to its considerable contents of polar amino acids, such as glutamic and aspartic acid [26].

The superabsorbent capacity of protein-based materials is strongly dependent on the processing conditions, being reduced as temperature increases due to the promotion of crosslinking [5,27]. Furthermore, longer residence times, either in the mold or during storage at relatively high temperatures, lead to an increase in the physical crosslinking within the structure, also hindering the swellability of the samples [5,10,28]. Likewise, the variation of the pH in the raw protein material affect the amount of water that samples can hold [6]. Temperature, time, and pH are crucial factors, as they promote changes in the sample structure, and consequently in the existing interactions between the protein chains [29]. As mechanical properties are mostly inversely related to water absorption, superabsorbent materials commonly possess very poor mechanical properties, sometimes even being solubilized to a certain extent when immersed [5,10]. In an attempt to overcome this drawback, certain strategies have been pursued (e.g., crosslinking agents, acrylic co-polymerization), although at the cost of their ecological character [4,30]. The solubilization and freeze-drying of proteins might impact their conformational structure [31], as has been highlighted before when this procedure was used to modify the pH of PPP [6]. The alteration of the molecular structure through freeze-drying could eventually influence the properties of the material that would be obtained from that protein source after thermal processing (i.e., injection molding) [32,33,34], even though the economic and environmental impacts should not be neglected if applied industrially.

The present manuscript aims to study the effects of freeze-drying on the properties of porcine-plasma-based superabsorbent materials. For this purpose, the protein source samples are either directly freeze-dried or first solubilized in water and subsequently freeze-dried. To evaluate the differences, rheological measurements, mechanical and water uptake tests, and scanning electron microscopy are carried out.

## 2. Materials and Methods

### 2.1. Materials and Sample Preparation

In the present study, porcine plasma protein (PPP) was used as the raw material. The protein flour was kindly supplied by Proanda S.A (AproPork, Essentia Protein, Ankeny, IA, USA), asp: 6.97 g/100 g of protein; Glu: 10.04 g/100 g of protein. The PPP protein content (74% *w*/*w*) was determined by multiplying the percentage of nitrogen by 6.25 (the Kejdhal factor for this kind of material). The nitrogen content was estimated using a LECO CHNS-932 nitrogen analyzer (Leco Corporation, St. Joseph, MO, USA). The moisture content was estimated to be around 6% and the ash content was around 17%. Pharma-grade glycerol (Gly), delivered by Panreac Química S.A (Barcelona, Spain), was employed as a plasticizer for all systems.

Untreated PPP flour (UF) in as-received condition (6% humidity) was employed as the reference. Then, the effects of freeze-drying were studied through two different procedures: an initial procedure where the flour was conveniently frozen at −40 °C, then freeze-dried at −80 °C (FD) in a LyoQest freeze-dryer (Telstar Technologies, Barcelona, Spain); and a second procedure where 10 g of PPP was solubilized in 100 mL of deionized water, after which the PPP solution was frozen at −40 °C and subsequently freeze-dried at −80 °C (SFD).

Superabsorbent materials based on PPP have previously been obtained by performing a mild injection molding procedure [5,6,7], which was also followed in the present study. This procedure started with a blending stage in a Haake Polylab QC two-blade counter-rotating mixer (ThermoHaake, Karlsruhe, Germany), whereby 65 g of PPP and glycerol were intimately mixed at a 50:50 ratio. This stage took place at room temperature for 5 min and at 50 rpm, while the mixing rheometer recorded the torque and temperature in the mixing cavity. Subsequently, 1.5 g of the obtained homogeneous blend was injection molded into a rectangular mold (1 × 10 × 60 mm^3^) using a Minijet Piston Injection Molding System (ThermoHaake, Karlsruhe, Germany). The temperature of the feed cylinder was always 40 °C, while the mold temperature was 60 xB0;C and the pressure employed during the injection and holding stages, which lasted 150 s, was 500 bar.

### 2.2. Methods

#### 2.2.1. Linear Viscoelastic Properties

Viscoelastic properties were estimated using dynamic mechanical temperature analysis (DMTA) within the linear viscoelastic range (LVR) by carrying out compressional and torsional measurements for the blends and protein-based materials, respectively. A RSA3 rheometer (TA Instruments, New Castle, DE, USA) was used to perform the compression mode tests on blends using a cylindrical geometry measuring 8 mm in. diameter. On the other hand, protein-based materials were tested in a DHR-3 rheometer (TA Instruments, New Castle, DE, USA) in torsion mode. In every case, strain sweep tests (0.001–10%) were initially carried out at 1 Hz to identify the strain amplitudes that defined the LVR. Afterwards, temperature ramp tests were performed by employing a heating rate of 5 °C/min from 25 °C to 140 °C for blends or from −30 °C to 140 °C for the protein-based materials, at a constant frequency (1 Hz) and strain (within the LVR).

#### 2.2.2. Tensile Properties

In order to estimate the mechanical properties of the plastic samples, uniaxial tensile tests were performed until breaking point using a Dynamic Mechanical Analyser RSA3 (TA Instruments, New Castle, DE, USA), with a rectangular tensile geometry (tension mode) at a constant strain rate of 1 mm·min^−1^ at room temperature (≃25 °C). Typical mechanical stress-strain curves were obtained, from which mechanical properties were determined, such as the Young’s modulus (E), maximum or ultimate stress (σ_max_), and strain at break (ε_max_).

#### 2.2.3. Differential Scanning Calorimetry (DSC)

DSC tests were performed in an 822 calorimeter (Mettler Toledo, Worthington, OH, USA) using Mettler Toledo Star System software. For this purpose, 12–14 mg of biomass were located in hermetically sealed aluminum pans and tests were run at a rate of 10 °C/min from −25 to 300 °C using an empty pan as a reference.

#### 2.2.4. Water Uptake

Water uptake capacity (WUC) values for the obtained samples were determined using a protocol described in previous studies [1,5]. First, the protein-based materials were placed in an oven at 50 °C until constant weight (w_1_). Then, they were immersed in deionized water for 24 h and then weighed (w_2_). Finally, samples that had been dried for 24 h were weighed again (w_3_). The WUC and soluble matter loss (SML) can be calculated using the following equations [5]:

(1) $$\mathrm{WUC}\;\left(\%\right)=100\cdot \frac{\left(w_{2}-w_{3}\right)}{w_{3}}$$

(2) $$\mathrm{SML}\;\left(\%\right)=100\cdot \frac{\left(w_{1}-w_{3}\right)}{w_{1}}$$

#### 2.2.5. Scanning Electron Microscopy

Following immersion, swollen PPP-based samples were then freeze-dried (−80 °C, 0.01 mbar) and cut into small pieces (2–3 mm). Afterwards, they were gold-coated and observed using scanning electron microscopy (SEM). A ZEISS EVO (Carl Zeiss Microscopy, White Plains, NY, USA) microscope was used to evaluate the microstructure of the selected swollen PPP-based materials. Micrographs were acquired using a beam current of 11–12 pA at a working distance of 6 mm and with an acceleration voltage of 10 kV. Analyses were carried at 60x magnification. In addition, the pore size was studied using a digital processing software (ImageJ, Bethesda, MD, USA). The mean diameter was obtained by measuring several pores in the images obtained for each system.

### 2.3. Statistical Analysis

In the current study, all measurements were performed in triplicate. Statistical studies were performed using ANOVA comparisons in the Statgraphics software (The Plains, VA, USA). Uncertainty was expressed as mean values ± standard deviations, which were plotted for all calculated parameters.

## 3. Results and Discussion

### 3.1. Mixing Stage

**Figure 1:**
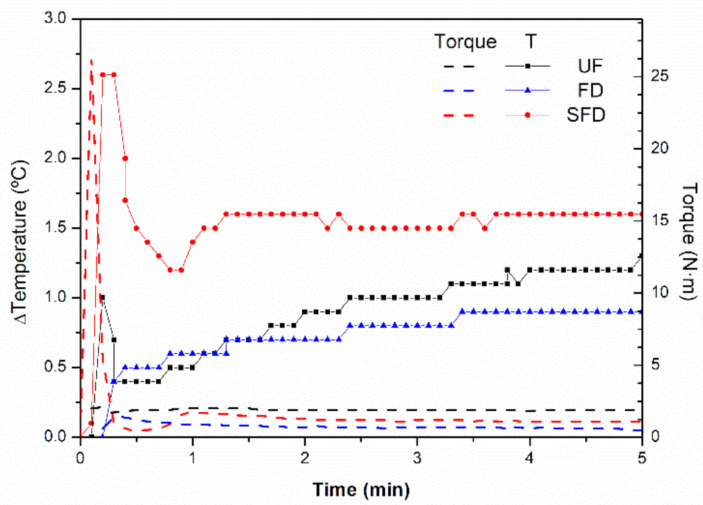
Torque and temperature profile developed within the mixer cavity during the mixing stage for porcine plasma protein-glycerol blends using UF, FD, and SFD protein systems.

In this section, the torque and temperature evolution inside the mixer as the PPP and glycerol were blended are presented in Figure 1. The evolution of both parameters was similar to that observed in previous studies for analogous systems [5,7]. The torque profile initially displayed a sudden increase due to the instantaneous compaction of the raw materials when pressed down by the plunger. The torque value dropped down to 2 N·m immediately after, remaining steady at this value during the whole mixing stage. Regarding the temperature profile (ΔTemperature), explained as the difference between the instantaneous temperature inside the mixer and the initial temperature of the blend, no noticeable changes were observed, as the largest increase of temperature recorded within the cavity of the mixer was about 2.5 °C for the SFD sample, remaining at 1.2 ± 0.3 °C at the end of the mixing stage in all cases. All samples showed the same tendency, which led to the conclusion that no significant mechanical energy dissipation took place inside the mixer during the mixing stage. This means that no important protein reticulation or crosslinking may be expected, as these interactions, which commonly occur when the processing conditions are extreme, typically involve an apparent increase in temperature [35].

### 3.2. Thermal Characterization of the Systems

#### 3.2.1. Evolution of the Rheological Properties of the Blends with Temperature

**Figure 2:**
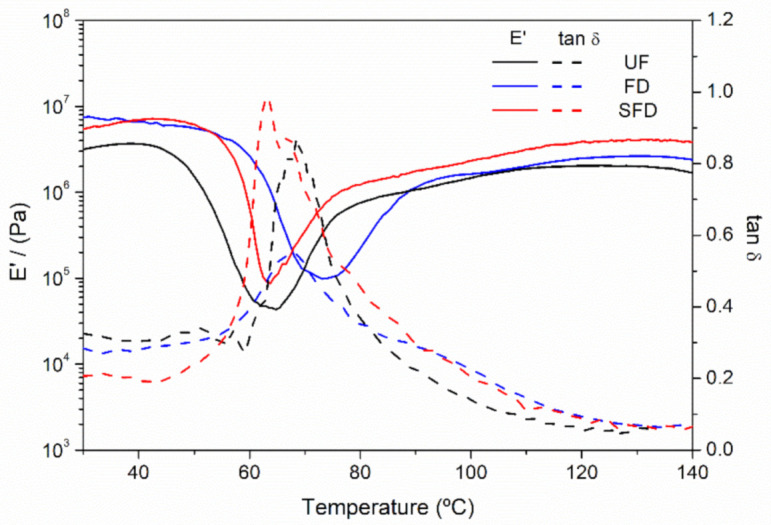
Evolution of the compressional storage modulus (E′) and loss tangent (tan δ) of blends from porcine plasma protein (PPP) and glycerol materials using UF, FD, and SFD protein systems, obtained through temperature sweep tests ranging from 30 to 140 °C at 1 Hz within the lineal viscoelastic range.

The homogeneous blends obtained just after the mixing stage were rheologically characterized (Figure 2) in order to observe the thermal transitions of the PPP systems, which provided valuable information for the subsequent injection procedure [7]. All studied samples showed similar behavior, presenting the same qualitative events: (I) at relatively low temperatures, steady storage moduli (E′) values were observed until a certain temperature (between 45 and 60 °C) was reached; then (II), a temperature-induced drop took place until a minimum was achieved, reaching a decrease of two orders of magnitude in the E′ values; from the temperature where the minimum was located, (III) an increase in E′ took place due to protein aggregation and gelation processes.

The glassy plateau observed in the first stage (I) was kept until a certain temperature, which seemed to be displaced onto higher temperatures when PPP samples were submitted to a freeze-drying step. Thus, the storage moduli of the reference sample (no freeze-drying) started to decrease around 45 °C, while both freeze-dried samples started to decrease at approximately 57 °C, independently of the procedure followed. Likewise, the freeze-drying of the protein flour also seems to quantitatively influence the viscoelastic moduli, showing lower values in the reference (UF) sample than for samples that were freeze-dried, regardless of having been previously solubilized in deionized water (SFD) or not (FD). The observed differences may be associated with the fact that the freeze-dried samples did not contain any moisture, unlike the reference, which contained 6% water, which could play a plasticizer role. Therefore, in spite of all samples possessing the same glycerol content, FD and SFD samples contained lower overall quantities of plasticizer, resulting in reduced mobility between chains, and eventually promoting greater viscoelastic moduli [36]. Moreover, the SFD PPP system could also have been affected by the difference in the ice nucleation history, which may have promoted a difference in the stresses exerted on the protein as the water removal gradually increased the protein concentration in the aqueous solution. As it was freeze-dried, a solution with increasing viscosity was formed, which could increase intermolecular reaction rates, resulting in an alteration in the protein conformation [37].

The decrease observed in the second stage (II) of the thermal treatment of the blends was reported to be associated with the promotion of the mobility of polymeric chains at higher temperatures [6]. At the end of this event, an apparent minimum was observed at a temperature that depended on the procedure followed, as previously observed for the glassy plateau. The reference sample displayed lower viscoelastic moduli values at the minimum point than the freeze-dried samples, which may be connected with the higher amount of plasticizer, as previously mentioned. These minimum values were located at 62.5 °C for the UF and SFD samples and at approximately 73 °C for the FD sample.

The increase in the storage modulus after the minimum (III) occurred as proteins such as albumin [38,39] aggregated. The two different slopes shown during the strengthening may correspond to the different protein fractions present in the PPP [5]. Additionally, similar behavior was presented in previous studies for PPP-Gly blends [5,6]. However, the effects of freeze-drying observed on the rheological properties of the protein source were quite apparent and innovative.

Additionally, as observed in Figure 2, the loss tangent (tan δ = E″/E′) generally showed values below the unity level across the whole temperature range studied, indicating a solid-like behavior for all samples, as the storage moduli (E′) were always higher than the viscous moduli (E″) [7,40]. A remarkable peak in tan δ could always be distinguished, which is typically referred to as the glass transition of the system [41]. Thus, the temperatures at which this peak occurred for the studied samples were between 60 and 65 °C, matching values previously reported for similar samples [6].

#### 3.2.2. Differential Scanning Calorimetry (DSC)

**Figure 3:**
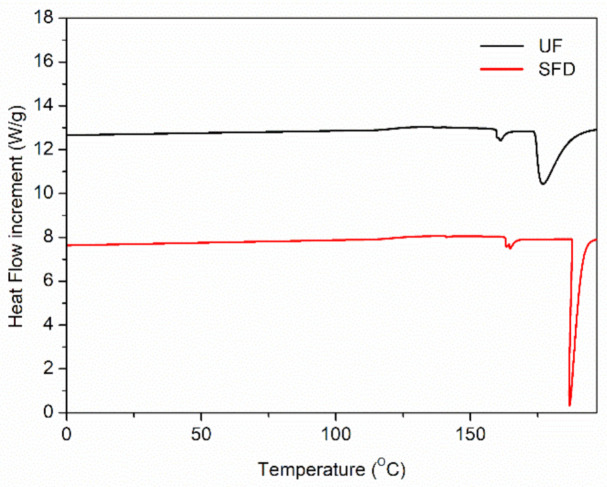
DSC thermograms for the UF and SFD porcine plasma protein systems run at a heating rate of 10 °C/min.

It should be highlighted that the thermal transitions detected in the SFD sample happened at higher temperatures but within a smaller temperature range than the rest. To confirm this, DSC was performed on both the UF reference and the SFD sample (Figure 3). DSC tests confirmed this, which could be explained by certain molecular rearrangements that might have taken place when dispersing the sample in deionized water.

Calorimetric techniques are widely used to identify the thermal transitions in proteins and other biomacromolecules [42,43]. Regarding these results, only two well-differentiated peaks could be distinguished in the thermograms—the first at around 162 and 167 °C and the second located at 177 and 187 °C for the UF and the SFD samples, respectively. The thermal energy values related to the first peak were around 2.4 (W/g)·°C and were quite similar for both samples. On the other hand, in the case of the second endothermal peak, it was slightly higher in the case of the solubilized and freeze-dried sample (27.5 (W/g)·°C) when compared to the reference (23.7 (W/g)·°C). These peaks might correspond to a denaturation point that would favor a greater flowability [44] that would take place in a broader temperature range for the reference (20 °C) than for the SFD sample (10 °C), with a thinner peak. Therefore, these results would confirm the fact that the SFD-containing blend showed thermal transitions across a smaller temperature range, just as observed in the DMTA tests (Figure 2). The differences among samples may be caused by the conformational changes suffered by the proteins after being solubilized in water, which could eventually produce alterations in the protein functionality and stability [45,46].

#### 3.2.3. Evolution of the Rheological Properties of the PPP-Based Materials with Temperature

**Figure 4:**
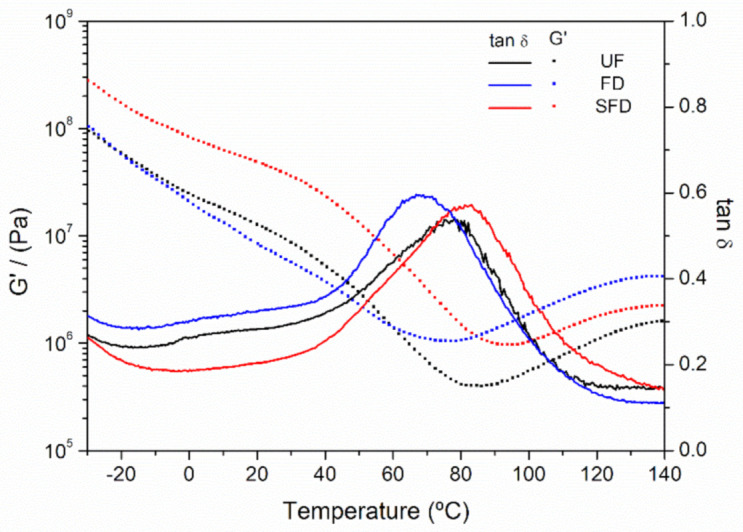
Evolution of the storage (G′) and viscous (G″) moduli in torsion mode for porcine plasma protein-glycerol materials using UF, FD, and SFD protein systems, obtained through temperature sweep tests from 30 to 140 °C at 1 Hz within the lineal viscoelastic range.

Injection-molded plastic materials obtained from UF, FD, or SFD systems were submitted to temperature sweep tests in order to identify the influence of temperature on their viscoelastic properties (Figure 4).

As expected, in all cases, the samples showed G′ values higher than those for G″, which resulted in tan δ values below the unity level across the whole temperature range. At lower temperatures, the sample subjected to the SFD process showed higher values for G′ than the rest of the samples, implying the formation of a more strengthened structure, which could be associated with the rearrangements that might have occurred during the solubilization process. As the temperature increased, softening was detected for all samples through a decrease in G′ until a minimum was found, from which G′ significantly increased. This increase in the viscoelastic properties took place at temperatures higher than 60 °C (the molding temperature), as previously reported for similar materials based on PPP and glycerol [5,6,7,47]. The increase in the viscoelastic properties as the temperature gets higher is typical of the thermosetting potential shown by plastic materials molded in relatively mild conditions, under which plasticized polymers still display thermoplastic behavior. A previous study highlighted the importance of mild processing conditions in producing superabsorbent materials from PPP, as thermal crosslinking hinders water uptake [5]. However, the promotion of the hydrophilic character of the materials is achieved at the expense of a poor strengthening of the structure, which sometimes leads to undesired disintegration of the superabsorbent material when immersed. Moreover, the UF sample displayed the lowest G′ values at the minimum point, which may be explained by the greatest plasticization degree being achieved due to the higher moisture content of the flour. However, samples obtained from freeze-dried flours displayed similar values for G′ at the minimum point, as water was removed from both of them. As the temperature further increased, a tendency toward plateau values was observed for all samples. Nevertheless, the FD sample finally displayed higher values of G′ than the SFD sample, indicating a greater thermosetting potential for this sample.

### 3.3. Mechanical Characterization of the PPP-Based Materials

**Figure 5:**
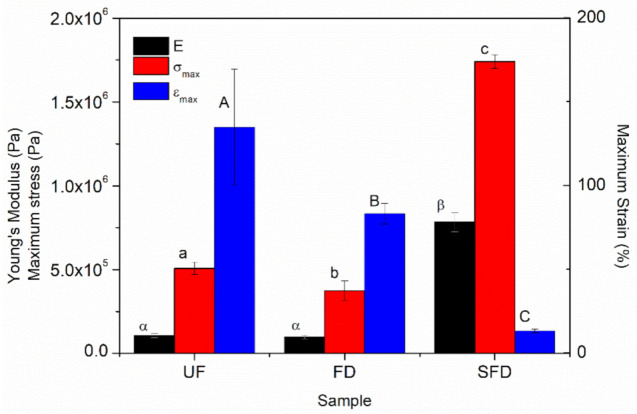
Mechanical parameters of porcine plasma protein-glycerol materials using UF, FD, and SFD protein systems, obtained through uniaxial tensile tests at a deformation rate of 1 mm/s. Average values marked with different lower-case or upper-case Greek letters are statistically different (*p* < 0.05).

The evolution of the main mechanical properties (E, σ_max_, ε_max_) of the protein-based materials obtained from PPP submitted to different procedures is shown in Figure 5. Typical stress-strain curves of uniaxial stress until breaking point were obtained for all samples. At the beginning of the curves, a linear slope characteristic of an initial Hookenian behavior could be distinguished, from which Young’s modulus (E) values could be determined. After the yield stress was surpassed, plastic deformation was observed, whereby small stresses resulted in important deformations. The tests ended when materials reached the ultimate stress point (σ_max_) and underwent rupture at the maximum strain point (ε_max_).

**Figure 6:**
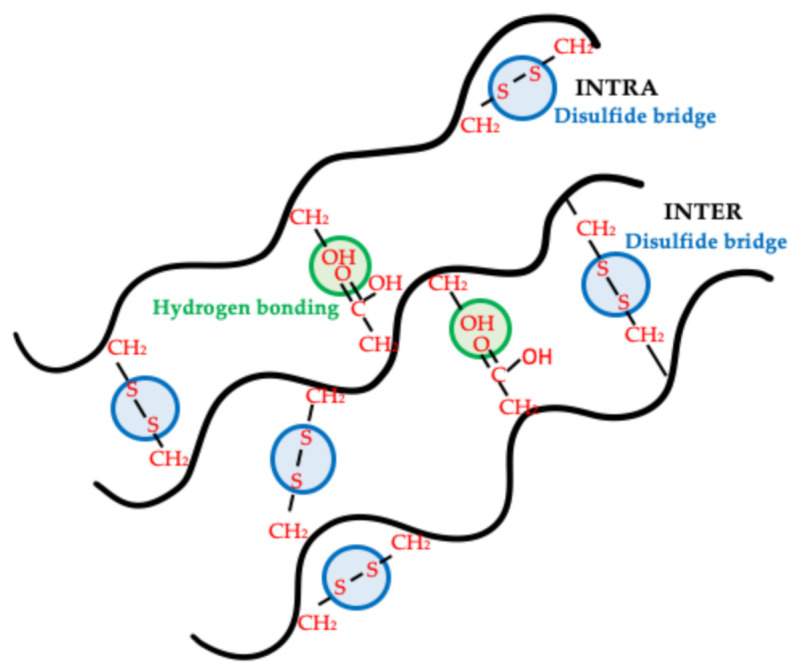
Proposed scheme for the main interactions promoted when protein unfolding takes place.

When the mechanical parameters of the materials obtained from the UF sample were compared to those of the materials from the FD source, no differences were perceived in terms of Young’s modulus values. However, a significant although slight decrease could be perceived in σ_max_ values, being more noticeable than ε_max_ values. The lower deformability shown by the FD sample might be related to the reduction in the amount of plasticizer (glycerol + moisture) [48]. On the other hand, more remarkable differences were determined for materials processed from the SFD flour. The SFD stage led to a greater strengthening of the injection-molded sample, as E increased from 0.1 × 10^5^ to 7.8 × 10^5^ Pa. This strengthening was also denoted by a remarkable increase in the tensile strength, as σ_max_ increased from 0.5 × 10^6^ to 1.8 × 10^6^ Pa. As the lower plasticizer content did not have a strong influence on E or σ_max_, the solubilization in deionized water should be the main reason for this reinforcement. Moreover, ε_max_ dropped from around 135% to 13% when PPP was solubilized, as the reinforcement made the samples more fragile. Some authors have reported that ice formation during freezing may promote protein denaturation through ice-protein interactions, altering the conformational structure of the protein [49]. Zhan et al. showed that unfolding takes place in proteins when they are freeze-dried, which may produce higher amount of reactive sites, promoting the bonding between chains (hydrophobic interactions and hydrogen bonding) (Figure 6) [50]. The results obtained with SFD samples seem to support this hypothesis, whereby a greater exposition of reactive groups along the polymeric chain to ice may lead to a greater reinforcement during the material processing.

### 3.4. Water Uptake Capacity of PPP-Based Materials

**Figure 7:**
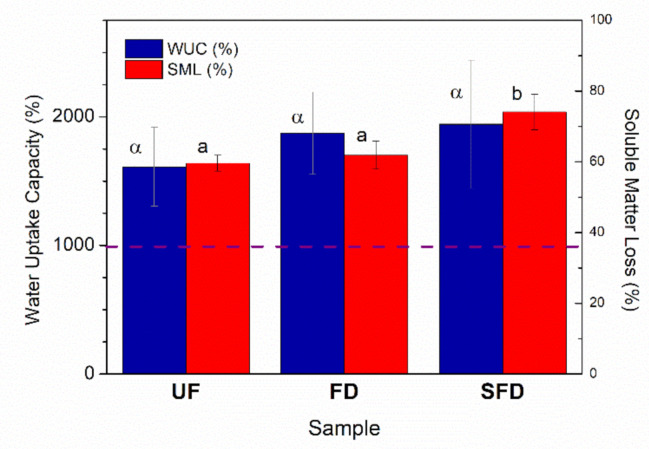
Water uptake capacities of porcine plasma protein-glycerol materials using UF, FD, or SFD protein systems, obtained through deionized water immersion over 24 h. The dashed line indicates the superabsorbent threshold. Average values marked with different lower-case Greek letters are statistically different (*p* < 0.05).

The water uptake capacity (WUC) values obtained for the different samples can be observed in Figure 7. Regarding the WUC values of the different samples obtained from different procedures (UF, FD, SFD), it is remarkable that all of them can be considered superabsorbent materials, as their WUC values surpassed the lowest threshold required (1000%) [2]. Superabsorbent materials were previously obtained from porcine plasma protein, as reported in some studies [5,6,7]. Furthermore, although the SFD sample displayed a higher WUC value than FD or UF samples, no significant differences were found. Thus, the reinforcement in the material achieved by the aqueous solubilization of PPP, as shown by the remarkable increases in the mechanical properties of the samples, did not seem to have any negative consequence in terms of the water absorption capacity.

Otherwise, the SFD treatment of the PPP prior to its blending and subsequent injection molding seemed to have a great impact on the soluble matter loss.

### 3.5. Scanning Electron Microscopy (SEM)

**Figure 8:**
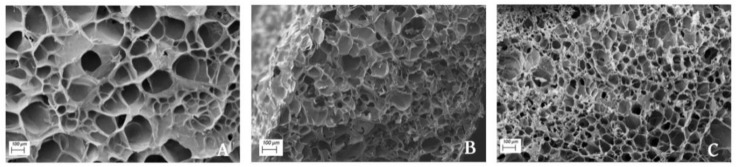
SEM micrographs of matrices obtained after swelling and freeze-drying of injection-molded reference (**A**), FD (**B**), and SFD (**C**) porcine plasma protein samples.

Figure 8 shows the micrographs obtained through SEM of the swollen and freeze-dried matrices of the reference sample (Figure 8A) and the samples submitted to the FD (Figure 8B) and SFD procedures (Figure 8C). The porous structure observed for all samples was caused by the inclusion of water into the polymeric structure during the immersion stage, as glycerol was lost into the immersion media during the 24 h immersion process. This entrapped water was later removed in the freeze-drying stage that took place after swelling, leading to the formation of pores throughout the structure. As can be seen, the UF sample had much larger pores (182 ± 53 μm) than the SFD sample (74 ± 15 μm), which displayed a larger number of smaller pores, while the FD sample contained intermediate pores (110 ± 30 μm). Thus, smaller pore sizes may be the result of the mentioned reinforcement in the structure, as shown by the increases in E and σ_max_ values [7,10] in Figure 5. As mentioned before, a conformational change should take place solubilized and freeze-dried proteins [31], which would influence the protein-protein interactions [42], and consequently the overall structure of the materials. In previous studies, reductions in pore size were achieved through an increase of the mold temperature or through an excessively long molding stage, causing thermal crosslinking, and consequently lower WUC values [5,10]. In the present manuscript, the obvious reduction the pore size was caused by the initial treatment of the raw material, which did not hinder the WUC but did improve the mechanical properties of the final material.

## 4. Conclusions

The solubilization and freeze-drying processes used in the development of green superabsorbent materials based on porcine plasma protein and glycerol seems to exert a significant influence on the final physicochemical properties.

When the protein source was only freeze-dried prior to blending with the plasticizer, slight changes in the rheological and mechanical properties could be detected, being mainly attributed to its lower plasticizer content due to moisture removal. Otherwise, the addition of a solubilization stage of the porcine plasma protein prior to freeze-drying resulted in greater differences. In this case, samples showed greater viscoelastic moduli across the whole temperature range, either for blends or PPP-based materials. Furthermore, the Young’s modulus and maximum stress values of the solubilized-freeze-dried samples were greater, being around 7.5 and 3.5 times higher, respectively. On the other hand, the maximum strain values reduced more than 10-fold when compared to the rest of the samples, making them considerably more fragile. The observed change in the mechanical properties could be supported by the noteworthy decrease in the pore size of the solubilized-freeze-dried samples.

One of the most remarkable facts of the present study is that neither of the two treatments carried out (FD or SFD) led to any significant modification of the water uptake capacity of the UF-containing matrix, with samples surpassing in every case the lowest threshold required to be consider as superabsorbent materials. Thus, improvements in the mechanical properties of superabsorbent materials developed from porcine plasma protein and glycerol could be achieved without needing to use a stronger thermal treatment, submitting the protein source only to freeze-drying (especially if a previous solubilization stage had been previously conducted) before mixing and injection.

## Data Availability

All the results shown in the manuscript can be provided if requested from the corresponding author.

## References

[1] Cuadri A.A.A., Romero A., Bengoechea C., Guerrero A. (2018). The Effect of Carboxyl Group Content on Water Uptake Capacity and Tensile Properties of Functionalized Soy Protein-Based Superabsorbent Plastics. J. Polym. Environ..

[2] Cuadri A.A., Romero A., Bengoechea C., Guerrero A. (2017). Natural superabsorbent plastic materials based on a functionalized soy protein. Polym. Test..

[3] Ruiz-Hitzky E., Darder M., Fernandes F.M., Wicklein B., Alcântara A.C.S., Aranda P. (2013). Fibrous clays based bionanocomposites. Prog. Polym. Sci..

[4] Song W., Xin J., Zhang J. (2017). One-pot synthesis of soy protein (SP)-poly(acrylic acid) (PAA) superabsorbent hydrogels via facile preparation of SP macromonomer. Ind. Crops Prod..

[5] Álvarez-Castillo E., Bengoechea C., Rodríguez N., Guerrero A. (2019). Development of green superabsorbent materials from a by-product of the meat industry. J. Clean. Prod..

[6] Álvarez-Castillo E., Bengoechea C., Guerrero A. (2020). Effect of pH on the properties of porcine plasma-based superabsorbent materials. Polym. Test..

[7] Álvarez-Castillo E., Bengoechea C., Guerrero A. (2020). Composites from by-products of the food industry for the development of superabsorbent biomaterials. Food Bioprod. Process..

[8] Zohuriaan-Mehr M.J., Pourjavadi A., Salimi H., Kurdtabar M. (2009). Protein- and homo poly(amino acid)-based hydrogels with super-swelling properties. Polym. Adv. Technol..

[9] Fernández-Espada L., Bengoechea C., Cordobés F., Guerrero A. (2016). Protein/glycerol blends and injection-molded bioplastic matrices: Soybean versus egg albumen. J. Appl. Polym. Sci..

[10] Álvarez-Castillo E., del Toro A.J., Aguilar J.M., Bengoechea C., Guerrero A., Bengoechea C., Del Toro A., Aguilar J.M., Guerrero A., Bengoechea C. (2018). Optimization of a thermal process for the production of superabsorbent materials based on a soy protein isolate. Ind. Crops Prod..

[11] Álvarez-Castillo E., Del Toro A.J., Aguilar J.M., Guerrero A., Bengoechea C. (2019). Formation of soy protein-based superabsorbent materials through optimization o f a thermal processing. Afinidad.

[12] Capezza Villa A.J. (2017). Novel Superabsorbent Materials Obtained from Plant Proteins.

[13] Jin S.-K., Choi J.-S., Kim G.-D. (2021). Effect of porcine plasma hydrolysate on physicochemical, antioxidant, and antimicrobial properties of emulsion-type pork sausage during cold storage. Meat Sci..

[14] Álvarez C., Rendueles M., Díaz M. (2012). Production of porcine hemoglobin peptides at moderate temperature and medium pressure under a nitrogen stream. Functional and antioxidant properties. J. Agric. Food Chem..

[15] Del Hoyo P., Rendueles M., Díaz M. (2008). Effect of processing on functional properties of animal blood plasma. Meat Sci..

[16] Verheijen L.A., Wiersema D., Hulshoff P., de Wit J. (1996). Management of Waste from Animal Product Processing.

[17] Benitez B., Barboza Y., Bracho M., Izquierdo P., Archile A., Rangel L., Marquez E. (1999). Efecto del pH y concentración de las proteínas sobre la propiedad de gelación de la sangre animal. Rev. Cient. Fac. Cienc. Vet..

[18] Parés D., Toldrà M., Saguer E., Carretero C. (2014). Scale-up of the process to obtain functional ingredients based in plasma protein concentrates from porcine blood. Meat Sci..

[19] Hurtado S., Saguer E., Toldrà M., Parés D., Carretero C. (2012). Porcine plasma as polyphosphate and caseinate replacer in frankfurters. Meat Sci..

[20] Hurtado S., Dagà I., Espigulé E., Parés D., Saguer E., Toldrà M., Carretero C. (2011). Use of porcine blood plasma in “phosphate-free frankfurters”. Procedia Food Sci..

[21] Ramos-Clamont G., Fernández-Michel S., Carrillo-Vargas L., Martinez-Calderón E., Vázquez-Moreno L. (2003). Functional properties of protein fractions isolated from porcine blood. J. Food Sci..

[22] Nuthong P., Benjakul S., Prodpran T. (2009). Effect of some factors and pretreatment on the properties of porcine plasma protein-based films. LWT—Food Sci. Technol..

[23] Samsalee N., Sothornvit R. (2019). Development and characterization of porcine plasma protein-chitosan blended films. Food Packag. Shelf Life.

[24] Sothornvit R., Krochta J.M. (2005). Plasticizers in edible films and coatings. Innovations in Food Packaging.

[25] Nuthong P., Benjakul S., Prodpran T. (2009). Characterization of porcine plasma protein-based films as affected by pretreatment and cross-linking agents. Int. J. Biol. Macromol..

[26] García M.C., Torre M., Marina M.L.L., Laborda F., Rodriguez A.R., Garcia M.C., Torre M., Marina M.L.L., Laborda F., Rodriquez A.R. (1997). Composition and characterization of soyabean and related products. Crit. Rev. Food Sci. Nutr..

[27] Bourny V., Perez-Puyana V., Felix M., Romero A., Guerrero A. (2017). Evaluation of the injection moulding conditions in soy/nanoclay based composites. Eur. Polym. J..

[28] Jiménez-Rosado M., Bouroudian E., Perez-Puyana V., Guerrero A., Romero A. (2020). Evaluation of different strengthening methods in the mechanical and functional properties of soy protein-based bioplastics. J. Clean. Prod..

[29] Gómez-Heincke D., Martínez I., Stading M., Gallegos C., Partal P. (2017). Improvement of mechanical and water absorption properties of plant protein based bioplastics. Food Hydrocoll..

[30] Rathna G.V.N., Damodaran S. (2001). Swelling behavior of protein-based superabsorbent hydrogels treated with ethanol. J. Appl. Polym. Sci..

[31] Gong K.-J., Shi A.-M., Liu H.-Z., Liu L., Hu H., Adhikari B., Wang Q. (2016). Emulsifying properties and structure changes of spray and freeze-dried peanut protein isolate. J. Food Eng..

[32] Stärtzel P., Gieseler H., Gieseler M., Abdul-Fattah A.M., Adler M., Mahler H.-C., Goldbach P. (2016). Mannitol/l-Arginine-Based Formulation Systems for Freeze Drying of Protein Pharmaceuticals: Effect of the l-Arginine Counter Ion and Formulation Composition on the Formulation Properties and the Physical State of Mannitol. J. Pharm. Sci..

[33] Costantino H.R., Firouzabadian L., Wu C., Carrasquillo K.G., Griebenow K., Zale S.E., Tracy M.A. (2002). Protein spray freeze drying. 2. Effect of formulation variables on particle size and stability. J. Pharm. Sci..

[34] Schersch K., Betz O., Garidel P., Muehlau S., Bassarab S., Winter G. (2010). Systematic investigation of the effect of lyophilizate collapse on pharmaceutically relevant proteins I: Stability after freeze-drying. J. Pharm. Sci..

[35] Apichartsrangkoon A., Ledward D. (2002). Dynamic viscoelastic behaviour of high pressure treated gluten–soy mixtures. Food Chem..

[36] Perez-Puyana V., Felix M., Romero A., Guerrero A. (2016). Characterization of pea protein-based bioplastics processed by injection moulding. Food Bioprod. Process..

[37] Fang R., Bogner R.H., Nail S.L., Pikal M.J. (2020). Stability of Freeze-Dried Protein Formulations: Contributions of Ice Nucleation Temperature and Residence Time in the Freeze-Concentrate. J. Pharm. Sci..

[38] Aguilar J.M., Jaramillo A., Cordobés F., Guerrerro A. (2010). Influencia del procesado térmico sobre la reología de geles de albumen de huevo. Afinidad.

[39] Aguilar J.M., Cordobes F., Jerez A., Guerrero A. (2007). Influence of high pressure processing on the linear viscoelastic properties of egg yolk dispersions. Rheol. Acta.

[40] Zárate-Ramírez L.S., Romero A., Martínez I., Bengoechea C., Partal P., Guerrero A. (2014). Effect of aldehydes on thermomechanical properties of gluten-based bioplastics. Food Bioprod. Process..

[41] Bengoechea C., Arrachid A., Guerrero A., Hill S.E., Mitchell J.R. (2007). Relationship between the glass transition temperature and the melt flow behavior for gluten, casein and soya. J. Cereal Sci..

[42] Sedov I., Nikiforova A., Khaibrakhmanova D. (2020). Evaluation of the binding properties of drugs to albumin from DSC thermograms. Int. J. Pharm..

[43] Johnson C.M. (2013). Differential scanning calorimetry as a tool for protein folding and stability. Arch. Biochem. Biophys..

[44] Adebisi A.O., Kaialy W., Hussain T., Al-Hamidi H., Nokhodchi A., Conway B.R., Asare-Addo K. (2020). Freeze-dried crystalline dispersions: Solid-state, triboelectrification and simultaneous dissolution improvements. J. Drug Deliv. Sci. Technol..

[45] Pierson N.A., Makarov A.A., Strulson C.A., Mao Y., Mao B. (2017). Semi-automated screen for global protein conformational changes in solution by ion mobility spectrometry-massspectrometry combined with size-exclusion chromatography and differential hydrogen-deuterium exchange. J. Chromatogr. A.

[46] Silva J.L., Foguel D., Da Poian A.T., Prevelige P.E. (1996). The use of hydrostatic pressure as a tool to study viruses and other macromolecular assemblages. Curr. Opin. Struct. Biol..

[47] Álvarez-Castillo E., Oliveira S., Bengoechea C., Sousa I., Raymundo A., Guerrero A. (2020). A rheological approach to 3D printing of plasma protein based doughs. J. Food Eng..

[48] Felix M., Romero A., Cordobes F., Guerrero A. (2015). Development of crayfish bio-based plastic materials processed by small-scale injection moulding. J. Sci. Food Agric..

[49] Arsiccio A., Giorsello P., Marenco L., Pisano R. (2020). Considerations on Protein Stability during Freezing and Its Impact on the Freeze-Drying Cycle: A Design Space Approach. J. Pharm. Sci..

[50] Zhan F., Shi M., Wang Y., Li B., Chen Y. (2019). Effect of freeze-drying on interaction and functional properties of pea protein isolate/soy soluble polysaccharides complexes. J. Mol. Liq..

